# Cellular diamine levels in cancer chemoprevention: modulation by ibuprofen and membrane plasmalogens

**DOI:** 10.1186/1476-511X-10-214

**Published:** 2011-11-16

**Authors:** Paul L Wood, M Amin Khan, Tara Smith, Dayan B Goodenowe

**Affiliations:** 1 Dept. of Pharmacology, DeBusk College of Osteopathic Medicine, Lincoln Memorial University, 6965 Cumberland Gap Pkwy., Harrogate, TN 37752 USA; 2 R&D Dept., Phenomenome Discoveries Inc, 204-407 Downey Road, Saskatoon, SK, S7N 4L8 Canada

**Keywords:** CHO, NRel-4, ibuprofen, plasmalogens, omega-3 fatty acids, PPI-1011, putrescine, cadaverine, diamine exporter, ornithine decarboxylase, cancer chemoprevention

## Abstract

**Background:**

To develop effective strategies in cancer chemoprevention, an increased understanding of endogenous biochemical mediators that block metastatic processes is critically needed. Dietary lipids and non-steroidal anti-inflammatory drugs (NSAIDs) have a published track record of providing protection against gastrointestinal malignancies. In this regard, we examined the effects of membrane plasmalogens and ibuprofen on regulation of cellular levels of diamines, polyamine mediators that are augmented in cancer cells. For these studies we utilized Chinese hamster ovary (CHO) cells and NRel-4 cells, a CHO cell line with defective plasmalogen synthesis.

**Results:**

NRel-4 cells, which possess cellular plasmalogen levels that are 10% of control CHO cells, demonstrated 2- to 3-fold increases in cellular diamine levels. These diamine levels were normalized by plasmalogen replacement and significantly reduced by ibuprofen. In both cases the mechanism of action appears to mainly involve increased diamine efflux via the diamine exporter. The actions of ibuprofen were not stereospecific, supporting previous studies that cyclooxygenase (COX) inhibition is unlikely to be involved in the ability of NSAIDs to reduce intracellular diamine levels.

**Conclusions:**

Our data demonstrate that ibuprofen, a drug known to reduce the risk of colorectal cancer, reduces cellular diamine levels via augmentation of diamine efflux. Similarly, augmentation of membrane plasmalogens can increase diamine export from control and plasmalogen-deficient cells. These data support the concept that membrane transporter function may be a therapeutic point of intervention for dietary and pharmacological approaches to cancer chemoprevention.

## Background

While the focus of cancer research has largely involved the design of cytotoxic molecules, increasing efforts are being made to understand and utilize endogenous anticancer mechanisms. Areas of focus include dietary supplementation, identification of endogenous anticancer metabolites [1,2] and deregulation of polyamine catabolism [3,4]. Chemoprevention against colorectal [5-9] and prostate [10,11] neoplasia has been demonstrated with aspirin and some NSAIDs, like ibuprofen and sulindac, as well as with difluoromethylornithine (DFMO), an ornithine decarboxylase (ODC) inhibitor that decreases polyamine biosynthesis.

While aspirin and NSAIDs act to decrease local inflammation via cyclooxygenase (COX) inhibition, they also produce large decreases in the levels of the polyamines spermidine, putrescine and cadaverine, actions that are independent of COX inhibition [12]. Homeostatic regulation of intracellular polyamine and diamine levels is essential for normal cell growth and restriction of hyperplasia and neoplasia. Regulation of diamine levels is achieved by multiple points of physiological regulation. These include: i) the rate limiting enzyme ODC that converts ornithine to putrescine [13,14]; ii) polyamine metabolism by polyamine oxidase, spermine oxidase and spermine/spermidine acetyltransferase [15,16]; iii) polyamine uptake [17]; and iv) diamine export [17,18].

Complex changes in plasmalogens and fatty acid precursors of plasmalogens have been reported for cancer cells and the plasma of cancer patients [19]. Dietary omega-3 fatty acids, which are utilized in plasmalogen synthesis, are also known to decrease the risk of several cancers [20,21]. Decrements in plasmalogen levels, alterations in deacylation-reacylation of plasmalogens, and potential alterations in transport of plasmalogens, resulting from increases in scramblase 1, have all been reported to potentially contribute to neoplasia [22,23]. In this regard, we have studied plasmalogen deficiency in NRel-4 cells [24-26], a CHO cell mutant not expressing dihydroxyacetone-phosphate acyltransferase (EC 2.3.1.42), a peroxisomal enzyme essential for plasmalogen synthesis. These cells possess plasmalogen levels that are 5 to 10% of those measured in control CHO cells [25]. In a targeted metabolomics analysis utilizing four GC-MS panels that assay over 200 metabolic intermediates in amino acid, nucleotide, alcohol, sugar, polyol, fatty acid, and organic acid pathways [26], we observed that N-Rel cells had large increases in the intracellular diamines putrescine and cadaverine. In this study we report our findings regarding the effects of plasmalogen replacement [27] and of ibuprofen treatment on cellular levels of diamines, diamine synthesis, and diamine exporter function in CHO [28] and NRel [24] cells.

## Materials and methods

### Tissue Culture

CHO and NRel-4 cells (generous gift of Dr. R.A. Zoeller, Boston University) were cultured (10 cm^2 ^plates) in DMEM:F12 (Mediatech) supplemented with 10% FBS (Invitrogen) and 1% antibiotic/antimycotic (Invitrogen). Cells were grown at 37°C in a 5% CO_2 _incubator and treated with ibuprofen or PPI-1011, an ether lipid plasmalogen precursor, in DMSO at 80% confluence. At the conclusion of incubations, the wells were washed twice with cold phosphate buffered saline (PBS) and the plates harvested with versene/Trypleexpress and stored at -80°C until analyzed.

### Putrescine Release

Cells were incubated with DMSO (0.05% final), ibuprofen (10 μM) or PPI-1011 (50 μM) for 48 hr. Next the cells were washed with PBS and incubated in Hank's Balanced Salt Solution (HBSS) containing 700 μM arginine, to support cellular ornithine synthesis, and 15 mM HEPES (pH7.4) for 1.5 hr. The medium was collected, spiked with [^2^H_4_]putrescine, dried with a centrifugal evaporator and assayed for released putrescine. Cells were harvested and the released putrescine expressed as a percentage of the total cellular pool of putrescine.

### Putrescine Synthesis

Cells were incubated with 100 μM [^13^C_5_]ornithine in HBSS containing 700 μM arginine and 15 mM HEPES (pH7.4) for 30 min. Cells were washed with cold PBS and harvested as described above, prior to measurement of cellular [^13^C_5_]ornithine and [^13^C_4_]putrescine pools.

### Diamine Quantitation

Cells were sonicated in 1.2 ml of acetonitrile:MeOH:formic acid (800:200:2.4) containing [^2^H_4_]putrescine, [^2^H_4_]cadaverine and [^2^H_5_]ornithine (Cambridge and CDN Isotopes) as internal standards. For the putrescine synthesis experiments, [^2^H_4_]cadaverine was used as the internal standard. The cell lysates were transferred to 1.5 ml microtubes, sonicated and centrifuged at 4°C and 25,000 × g for 30 min. Next, 400 μL of the supernatant were dried in a centrifugal evaporator. To the dried cell extracts and the dried releasates were added 50 μL of pentafluorobenzyl (PFB) bromide solution (50 μL PFB-Br + 950 μL dimethylformamide) and 10 μL of diisopropylamine as catalyst. The samples were heated with shaking at 80°C for 1 hour and then vortexed with 200 μL of hexane/ethyl acetate (3:2). The tubes were then centrifuged at 25,000 × g for 5 min to precipitate salts. The supernatants were transferred to autosampler vials for GC-MS analyses.

The PFB derivatives were analyzed by ammonia NCI-GC-MS via monitoring the [M-181-3(HF)]^- ^anions for putrescine (567), [^2^H_4_]putrescine (571), cadaverine (581), and [^2^H_4_]cadaverine (585). The [M-181]^- ^anions were monitored for ornithine (671.1), [^2^H_5_]ornithine (676.2), lysine (658.1) and [^2^H_4_]lysine (689.1). All GC-MS analyses were performed with an Agilent 7890A GC and Agilent 5975C mass analyzer. The GC column was a 30 m HP-5MS (0.25 mm ID; 0.25 μm film).

### Immunocytochemistry

CHO and NRel cells were grown on glass coverslips (Thermo Scientific) until plates were approximately 50% confluent. Cells were then fixed by flooding coverslips with 4% paraformaldehyde in PBS for 10 minutes. Following two 5 min rinses in PBS the cells were blocked with 3% skim milk in 0.1% triton X100 PBS for 20 minutes. Cells were stained with an anti-SLC3A2 primary antibody (1:50, Santa Cruz Biotechnology) for 2 hours at room temperature. Excess antibody was removed by rinsing twice in PBS before exposing cells to labeled secondary IgG Alexa 594 antibody (1:400, Invitrogen) for 1 hr at room temperature. Coverslips where then rinsed twice in PBS before applying Hoescht 33258 for 10 min to stain all nuclei. Finally, cells were rinsed twice more in PBS before mounting in Prolong (Molecular Probes) and viewed by fluorescence microscopy.

### Data Analyses

Data are presented as mean ± SEM for 6 to 8 plates. Metabolite levels were expressed on a per mg protein basis. GC-MS analyses were performed using 5 point standard curves (reference standards at 0.2 to 10 times the stable isotope internal standard). Data were analyzed by 1-way ANOVA, followed by the Tukey-Kramer test for multiple comparisons.

## Results

### CHO and NRel Diamine Levels

**Figure 1:**
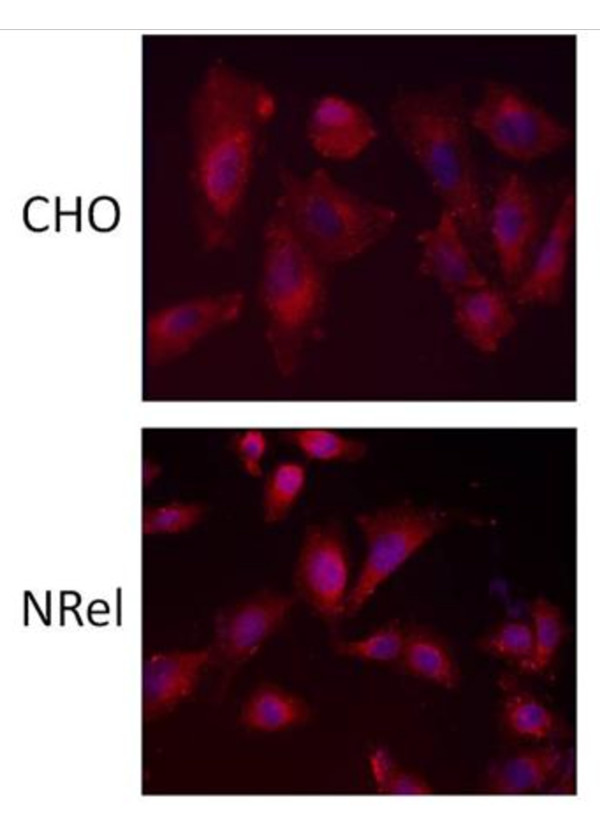
**Cultured CHO and NRel cells stained with an anti-SLC3A2 antibody (red) and Hoescht 33258 stain for nuclei (blue), at a 20× magnification**.

NRel-4 steady-state levels of the diamines putrescine and cadaverine were approximately 3-fold and 2-fold of CHO levels, respectively (Table 1). There was no difference in the levels of ornithine, the direct precursor of putrescine or in lysine, the direct precursor of cadaverine. Arginine levels also were the same in both cell lines. The diamine exporter, as visualized with antibodies to SLC3A2, was similarly expressed in the cell membrane of both CHO and N-Rel cells (Figure 1), as reported previously for CHO cells [18].

**Table 1:** Steady-state levels of amino acids and diamines in untreated CHO and N-Rel cells.

Parameter	CHO	N-Rel
Putrescine (nmol/mg protein)	2.06 ± 0.098	6.69 ± 0.74*

Cadaverine (pmol/mg protein)	123.1 ± 5.1	231.4 ± 27.0*

Ornithine (pmol/mg protein)	703.4 ± 73.4	682.0 ± 41.9

Lysine (nmol/mg protein)	15.1 ± 1.85	13.5 ± 0.90

Arginine (nmol/mg protein)	12.2 ± 0.49	11.6 ± 0.64

Data are presented as mean ± SEM (N = 8). *, p < 0.01

### Ibuprofen Effects

**Figure 2:**
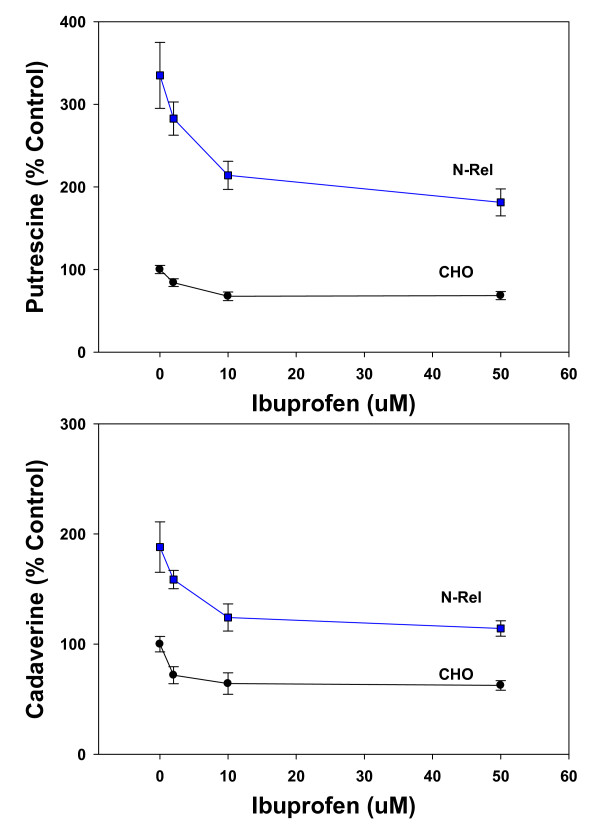
**Steep concentration-dependent decreases in cellular putrescine and cadaverine levels in CHO and NRel-4 cells incubated with ibuprofen (0-50 μM)**. Fresh medium and ibuprofen were provided at time 0 and at 24 hr. of a 48 hr. incubation. Decreases in putrescine and cadaverine levels, with 2, 10 and 50 μM ibuprofen, were significant (P < 0.05) for both CHO and NRel-4 cells. N = 6. Mean ± SEM.

**Figure 3:**
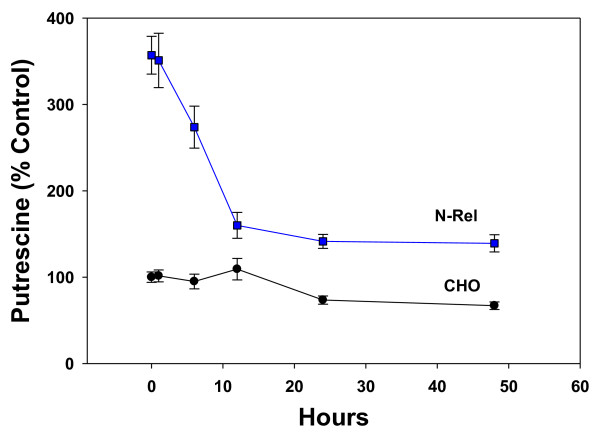
**Time course of decreases in cellular putrescine in CHO and NRel-4 cells during incubation with 50 μM ibuprofen**. N = 6. Mean ± SEM. Decreases were significant (p < 0.01) in NRel cells by 6 hr and by 24 hr in CHO cells.

**Figure 4:**
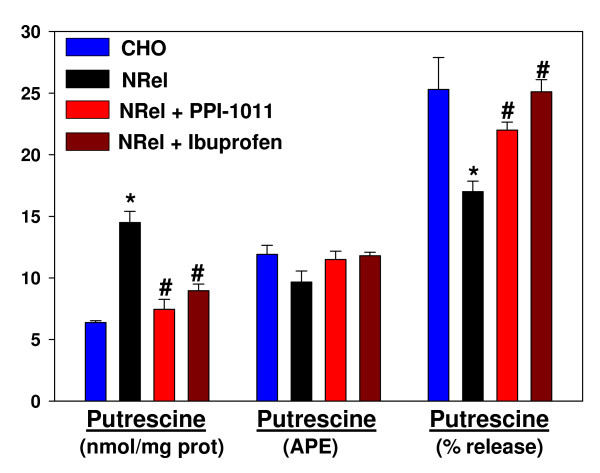
**Total cellular putrescine levels (nmol/mg protein); [^13^C_5_]ornithine decarboxylation to [^13^C_4_]putrescine, expressed as atom percent excess (APE); and levels of putrescine released into the medium, expressed as a percentage of the total cellular putrescine pool**. Labeling of cellular [^13^C_4_]putrescine with [^13^C_5_]ornithine (100 μM) was a 30 min. incubation. The intracellular ornithine pool was > 97% [^13^C_5_]labeled in all cells. Efflux of putrescine was measured using a 1.5 hr incubation. Drug concentrations were 10 μM (ibuprofen) and 50 μM (PPI-1011) for 48 hr. prior to the experiment. N = 6. Mean ± SEM. *, p < 0.01 vs. CHO; #, p < 0.01 vs. untreated NRel.

Incubations with ibuprofen were found to decrease diamine concentrations in a concentration- and time-dependent manner in both CHO and NRel cells. The concentration-response curves were very steep, with a maximum effect of 50% decreases in diamine levels (Figure 2). Time-course studies indicated that the actions were time-dependent, achieving maximum response by 24 hr in CHO cells but by 12 hr in NRel-4 cells (Figure 3). The ability of ibuprofen to lower cellular putrescine was demonstrated to lack stereospecificity (data not shown) and involved increased efflux of putrescine (Figure 4). Synthesis of putrescine from labeled ornithine was not altered in NRel-4 cells by ibuprofen treatment (Figure 4).

### Plasmalogen Effects

**Figure 5:**
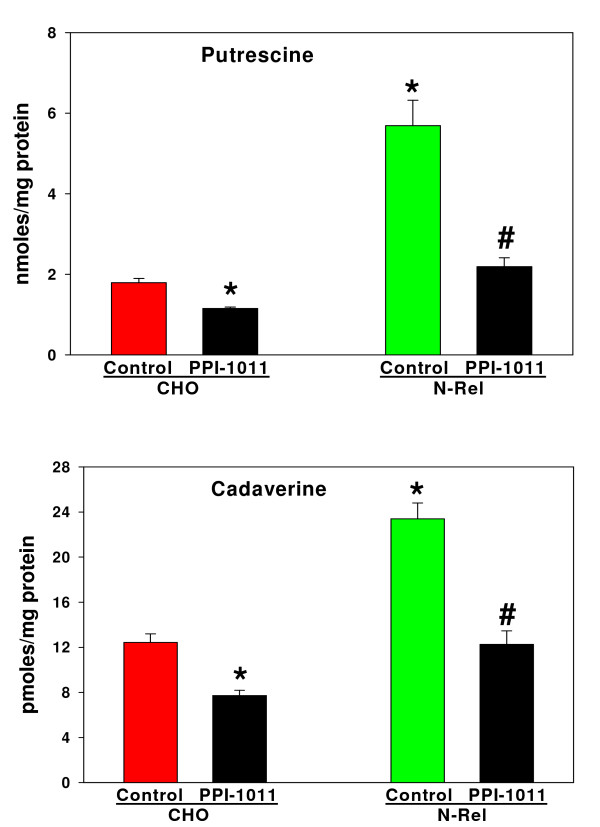
**Normalization of NRel-4 putrescine and cadaverine levels after a 72 hr incubation with PPI-1011 (100 μM)**. N = 6. Mean ± SEM. *, p < 0.01 vs. CHO; #, p < 0.01 vs. untreated NRel.

The cellular levels of diamines in NRel-4 cells were restored to normal levels (Figure 5) by augmenting plasmalogens with the ether lipid precursor, PPI-1011 [25,27]. Augmenting cellular plasmalogens in CHO cells also resulted in a small but significant reduction in cellular diamine levels (Figure 5). As with ibuprofen, PPI-1011 augmentation of cellular plasmalogens did not alter synthesis of putrescine from labeled ornithine (Figure 4) but did augment cellular export of putrescine (Figure 4).

## Discussion

Cancer chemoprevention strategies [29] represent a clinical approach to augmenting endogenous cytoprotective mechanisms to prevent cancer development and constitute an alternative approach to the never-ending search for the magic bullet that only kills cancer cells. Areas of focus in cancer chemoprevention currently include metabolomics of endogenous anticancer metabolites [1,2]; mechanistic studies of the established cancer prevention provided by regular aspirin or NSAID use [7]; studies of the roles and mechanisms of dietary omega-3 fatty acids in cancer prevention [20,21]; and down-regulation of polyamine synthesis to block cancer development [3-6,8,9,11,13].

**Figure 6:**
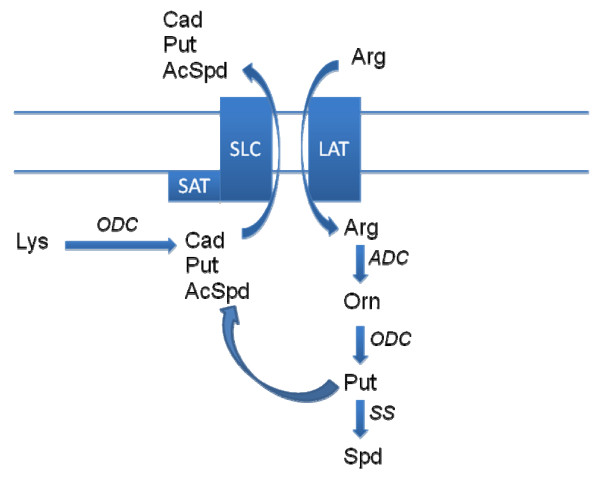
**Schematic representation of the diamine exporter**. AcSpd, acetylspermidine; ADC, arginine decarboxylase; Arg, arginine; CAD, cadaverine; LAT, L-amino acid transporter; ODC, ornithine decarboxylase; Orn, ornithine; Put, putrescine; SLC, SLC3A2 solute carrier; Spd, spermidine; SAT, spermidine/spermine N-acetyltransferase; SS, spermidine synthase.

Putrescine and cadaverine are diamines that have been shown to be increased in a number of cancer tissues, including cervical, colon, endometrial, oral cavity squamous cell, ovarian, pancreatic, and prostate [30-33]. Cellular studies have also demonstrated that a large fraction of polyamine metabolism is comprised of cellular export of putrescine [34] via the diamine exporter [17,18]. Similarly, the diamine cadaverine, which is generated by ODC metabolism of lysine (Figure 6), is exported via the diamine exporter [17,18,35]. Previous studies of the anti-cancer actions of aspirin have demonstrated that aspirin decreases polyamine synthesis at the level of ODC [12] and increases polyamine metabolism via induction of spermine/spermidine N-acetyltransferase [SAT; [36]]. With our studies of ibuprofen, we did not detect any drug effects on polyamine synthesis; however, we did measure increased cellular efflux of putrescine. In this regard, SAT has been shown to be complexed with the diamine exporter, thereby coupling polyamine acetylation and export [18]. Previous data demonstrating induction of SAT with aspirin [36] and our data demonstrating increased diamine exporter function with ibuprofen, suggest that augmentation of the diamine exporter -SAT membrane complex may be a mechanism of action that contributes to the cancer chemoprevention provided by aspirin and NSAIDs. Our data demonstrating that the actions of ibuprofen on cellular diamines are not stereospecific are consistent with previous publications demonstrating that the anti-cancer actions of aspirin [12] and NSAIDs [37] are independent of COX inhibition. These observations also suggest that safer analogs devoid of COX inhibition might be optimal drug candidates for cancer chemoprevention. Use of the ODC inhibitor, difluromethylornithine that reduces cellular polyamine levels is one such approach currently in clinical trials [8,11].

Our data also are the first to demonstrate modulation of the diamine exporter by docosahexaenoic acid (DHA)-containing ethanolamine plasmalogens. Augmentation of plasmalogens with the ether lipid precursor, PPI-1011 [25,27] decreased putrescine levels in CHO cells and normalized putrescine levels in NRel-4 cells to CHO cell levels. As with ibuprofen this appears to involve increased putrescine export to control intracellular diamine homeostasis. These data are consistent with previous reports demonstrating that decrements in membrane plasmalogens dramatically alter membrane function via alterations in membrane lipid rafts which in turn leads to deregulation of cholesterol transport [24,38], muscarinic membrane receptors [39] and β-adrenergic membrane receptors [40,41].

In summary, our understanding of the role(s) of polyamines in the chemoprevention of gastrointestinal malignancies continues to grow. Our data demonstrate the importance of the diamine exporter-SAT complex (Figure 6) in maintaining intracellular diamine levels and further show that a number of dietary and pharmaceutical approaches are available to provide protection against gastrointestinal malignancies.

## Competing interests

The authors are all involved in the preclinical development of PPI-1011.

## Authors' contributions

All authors read and approved the manuscript. All authors participated in the study design, supervision of assay QA/QC and data interpretation. TS and PW performed experiments.
